# Measurement of Twitch Dynamics in Response to Exercise Induced Changes in Mitochondrial Disease Using Motor Unit Magnetic Resonance Imaging (MUMRI): A Proof‐of‐Concept Study

**DOI:** 10.1002/nbm.70021

**Published:** 2025-04-09

**Authors:** Matthew G. Birkbeck, Mathew Elameer, Linda Heskamp, Jane Newman, Renae J. Stefanetti, Isabel Barrow, Oksana Pogoryelova, Gráinne S. Gorman, Julie Hall, Ian S. Schofield, Andrew M. Blamire, Roger G. Whittaker

**Affiliations:** ^1^ Newcastle University Translational and Clinical Research Institute (NUTCRI) Newcastle University Newcastle upon Tyne UK; ^2^ Northern Medical Physics and Clinical Engineering, Freeman Hospital Newcastle Hospitals NHS Foundation Trust Newcastle upon Tyne UK; ^3^ Department of Neuroradiology Royal Victoria Infirmary, Newcastle Upon Tyne NHS Foundation Trust Newcastle upon Tyne UK; ^4^ Wellcome Centre for Mitochondrial Research Newcastle University Newcastle upon Tyne UK; ^5^ National Institute for Health and Care Research Newcastle Biomedical Research Centre Newcastle upon Tyne UK; ^6^ NHS Highly Specialised Service for Rare Mitochondrial Disorders Newcastle Upon Tyne NHS Foundation Trust Newcastle upon Tyne UK

**Keywords:** exercise, fatigue, mitochondrial disease, motor unit, twitch dynamics

## Abstract

Muscle twitch dynamics and fatigability change in response to muscle disease. In this study, we developed an imaging paradigm to measure muscle twitch dynamics, and the response of the muscle to voluntary fatiguing contractions. We used a novel imaging technique called motor unit magnetic resonance imaging (MUMRI). MUMRI allows visualisation of muscle and motor unit activity by combining in‐scanner electrical stimulation with dynamic pulsed gradient spin echo (twitch dynamics, PGSE‐MUMRI) and phase contrast (fatigue, PC‐MUMRI) imaging. In Part I of this study, we scanned 10 healthy controls, we measured the muscle rise (T_rise_), contraction (T_contract_) and half‐relaxation time (T_half‐relax_) of the tibialis anterior (TA) muscle on a voxel‐wise basis using PGSE‐MUMRI. Five controls were scanned twice to assess reproducibility; PGSE‐MUMRI demonstrated reproducible results, with low variation between scans 3.4% for T_rise_, 6.4% for T_contract_ and 7.1% for T_half‐relax_. We then developed a PC‐MUMRI paradigm to measure the recovery of the TA in response to a fatiguing voluntary exercise. In Part II of the study, we applied these two novel imaging paradigms in a cohort study of nine patients with single large‐scale mtDNA deletion primary mitochondrial myopathy (PMM). Patients underwent a 12‐week resistance exercise programme and baseline, and follow‐up MRI was performed. PGSE‐MUMRI detected a significantly longer muscle contraction time between baseline and follow‐up in PMM patients 108.7 ± 7.9 vs. post‐119.3 ± 10.4 ms; *p* = 0.018. There was no statistical difference in the recovery half maximum measured using PC‐MUMRI in PMM patients between baseline and follow‐up 254 ± 109 vs. 137 ± 41 s; *p* = 0.074. In conclusion, PGSE‐MUMRI has detected differences in muscle twitch dynamics between controls and PMM following an exercise programme, and we can visualise differences in twitch dynamics subregions of muscle using this technique. The PC‐MUMRI technique has shown promise as a novel measure of muscle fatigue.

## Graphical abstract

Motor unit MRI (MUMRI) detected slower muscle contraction times in primary mitochondrial myopathies post resistance exercise programme and detected differences in muscle sub‐regions. This may evidence increased numbers of type 1 fibres post‐exercise. MUMRI could be used to measure changes in muscle twitch dynamics in neuromuscular diseases.

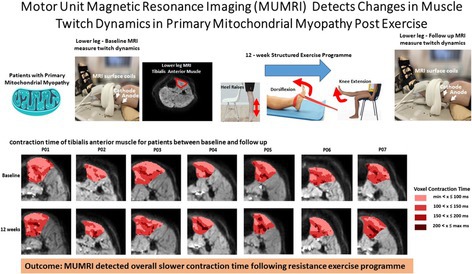

**Abbreviations**
ATP: adenosine triphosphate
DWI: diffusion weighted imaging
EMG: electromyography
MRS: magnetic resonance spectroscopy
MUMRI: motor unit magnetic resonance imaging
NHS: National Health Service
PC: phase contrast
PGSE: pulsed gradient spin echo
PMM: primary mitochondrial myopathy
REC: Research Ethics Committee
31P: 31 phosphorous

## 1 Introduction

Mitochondrial diseases are a heterogeneous group of genetic disorders affecting up to 1 in 4500 adults [1]. Perceived fatigue is one of the most common symptoms, affecting up to two thirds of all patients with mitochondrial disease [2]. However, it is important to differentiate between perceived fatigue and muscle fatigue, the latter is a quantifiable reduction in the ability to maintain an expected level of force during muscle contraction, which is also considered a common symptom of mitochondrial disease [3].

In recent years, several studies have investigated the potential of structured exercise programmes to improve muscle function. Endurance training improves oxidative capacity (a measure of how much oxygen a muscle can use at its maximum capacity [4]) in patients with heteroplasmic mtDNA variants [5]; whereas resistance training results in increased muscle strength and improved oxidative capacity in patients with a large‐scale single mtDNA deletion [6]. However, the further development of therapeutic exercise strategies is hampered by the limitations of currently available biomarkers to assess muscle function on an individual muscle level [7].

Clinical rating scales are typically subjective, non‐linear and relatively insensitive [8]. For example, the Borg scale rates effort and not a direct measure of muscle fatigability [9]. Exercise physiology such as measurement of peak aerobic exercise capacity are quantitative and relatively sensitive but provide no information on changes within individual muscles, while muscle biopsy is both invasive and samples a very limited volume of muscle [8, 10].

Dynamic serial phosphorus magnetic resonance spectroscopy (31P‐MRS) has been explored as an alternative biomarker [11]. This technique involves the use of an in‐scanner fatiguing protocol and serial MRS measurements of the recovery of the mitochondrial metabolite phosphocreatine. This provides a quantitative measure of mitochondrial bioenergetics and samples a far larger volume of muscle than a muscle biopsy. However, several disadvantages remain: there are difficulties in choosing the size and position of the sampling volume; the sampling volume typically encompasses several muscles precluding the analysis of individual muscles or muscle compartments. Furthermore, whilst 31P‐MRS measures the capacity of the mitochondrial respiratory chain, it provides no information on how this subsequently impacts the muscle's contractile properties [12]. PCr‐MR imaging gives higher spatial and temporal resolution than standard spectroscopic measures; however, all of these techniques require specialist 31P hardware and processing that are not routinely available [13].

We have recently introduced motor unit MRI (MUMRI) as a technique to assess the muscle's contractile properties. This has been described in detail elsewhere [14, 15, 16], but essentially, MUMRI allows non‐invasive imaging of individual human skeletal motor units. Two pulse sequences are currently employed: first, a diffusion weighted pulsed gradient spin echo (PGSE) sequence that is sensitive to contractile motion of the muscle fibres and can be used to image the size, shape and position of actively contracting muscle fibres [16]. This is extremely sensitive to muscle fibre contraction even at low motion encoding (b‐values). Second, using a bipolar gradient phase contrast sequence (PC), a quantitative measurement of motor unit twitch velocity can be derived [16]. By synchronising the scanner to a peripheral nerve stimulator and incrementally changing the latency between stimulation pulse and slice acquisition, the muscle contraction velocity can be plotted to produce quantitative twitch profiles covering the contraction and relaxation phases of motor unit activation on a voxel‐wise basis. This results in images with a resolution comparable with that acquired during conventional anatomical MRI [15].

We have previously applied these techniques to investigate muscle twitch dynamics across the spectrum of healthy ageing [14], demonstrating that the techniques were sensitive to determine differences between younger and older subjects, with older subjects having significantly longer muscle contraction times. In this study, we develop the MUMRI methodology to measure the muscle twitch dynamics following fatiguing voluntary muscle contractions; and then apply this protocol to measure changes in muscle function in patients with single large‐scale mtDNA deletion primary mitochondrial myopathy (PMM) before and after a structured exercise programme. We also further develop the analysis pathway to produce parametric maps which reveal information about muscle twitch dynamics in sub‐regions of individual muscles. This proof‐of‐concept study aims to develop a reproducible method of studying muscle twitch dynamics and compare it to current techniques such as 31P‐MRS.

## 2 Methods

**FIGURE 1:**
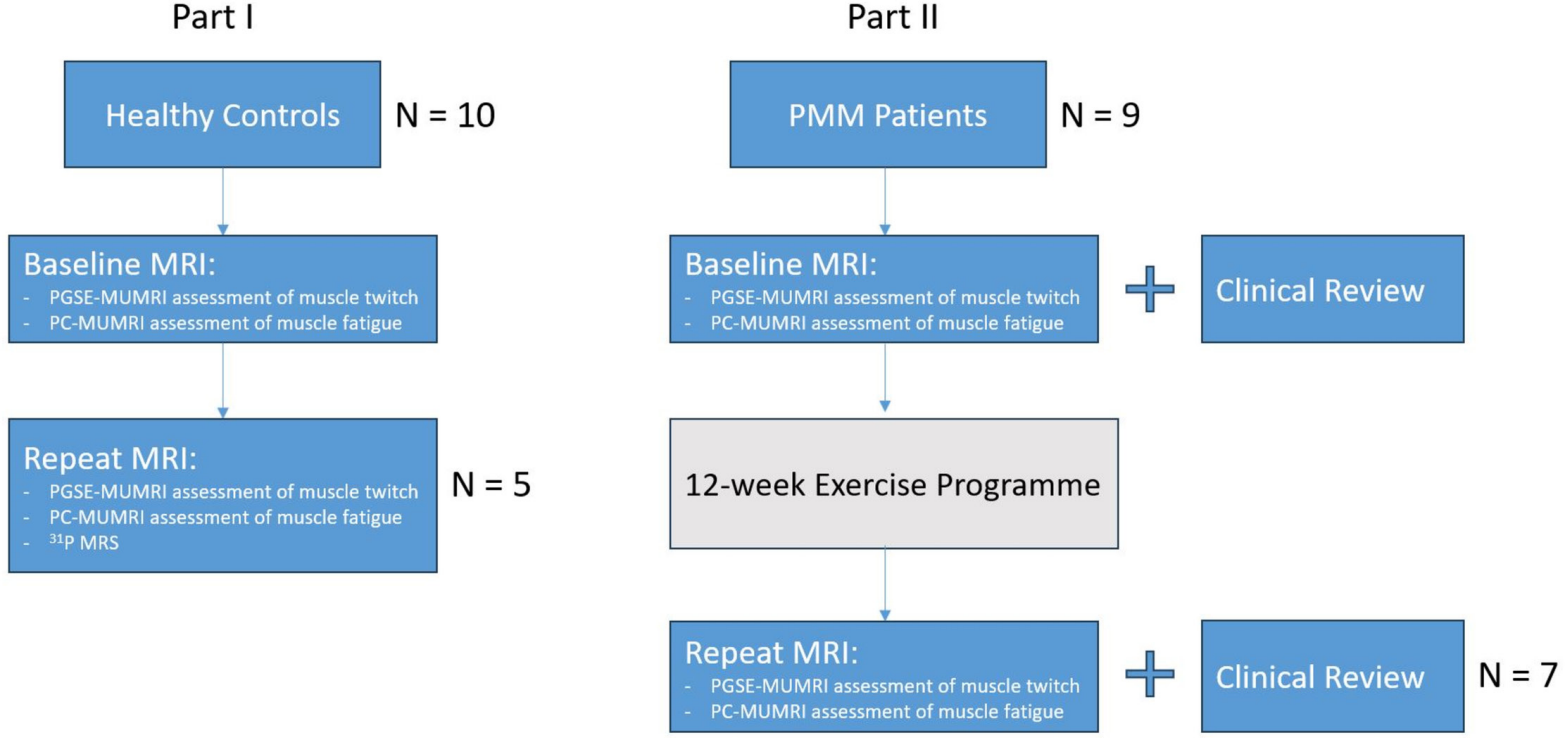
Flowchart providing an overview of the study design. Ten healthy controls underwent a baseline MRI, with a subset of five volunteers returning for reproducibility and 31P‐MRS comparison assessments. Then an interventional study in nine patients with PMM was performed, consisting of baseline clinical and imaging assessments followed by a 12‐week exercise intervention and subsequent follow‐up clinical and imaging assessments. Numbers of controls/patients shown at each stage.

This study consisted of two parts (Figure 1). In Part I, we developed a protocol combining MUMRI muscle twitch measurements with a muscle fatiguing isometric contraction protocol in a cohort of healthy adult volunteers. We examined inter‐session reproducibility and compared recovery of contractile kinetics measured with MUMRI with mitochondrial function assessed using 31P‐MRS. In Part II, we implemented this MUMRI fatigue protocol as a response measure in an interventional resistance exercise programme in patients with a confirmed diagnosis of PMM, with large scale single deletion. The muscle studied was the tibialis anterior as it is the primary muscle involved in dorsiflexion and can be easily stimulated using in‐scanner electrical stimulation [16, 17].

### 2.1 Part I: Establishing a MUMRI Fatigue Protocol in Healthy Subjects

Muscle fatigue occurs in response to prolonged or intense contractile activity and manifests as a decrease in the muscle's ability to generate force or power. This can result from a combination of biochemical and physiological factors [18], which vary by muscle fibre type and between health and disease [19]. With the onset of fatigue, individual motor units become unresponsive and lose their ability to undergo cycles of contraction and relaxation. MUMRI can be used to measure the twitch dynamics of motor units [14], through the use of electrical nerve stimulation and synchronised acquisition of either PGSE or PC images. As both sequences detect the contraction or relaxation of the muscle fibres within the stimulated motor units, we hypothesised that MUMRI can be used to quantify the recovery of the motor unit from exercise induced fatigue. In Part I, we implemented a measurement protocol using in‐scanner sustained isometric muscle contraction of sufficient magnitude to induce muscle fatigue followed, by stimulated MUMRI to measure the effect of the exercise on the muscle twitch.

### 2.2 Participants and Study Design

We recruited 10 healthy volunteers (mean ± SD, age: 45.8 ± 11.4 years) under ethics provided by Newcastle University (Ethical Approval Number: 1807/14592/2019). Written informed consent was obtained before the patient attended for the study visit. They were scanned on a 3T Philips Achieva X MRI scanner (Best, Netherlands) with a Diffusion Weighted Pulsed Gradient Spin Echo (PGSE‐MUMRI) sequence to assess the muscle twitch profile and Phase Contrast Motor Unit MRI (PC‐MUMRI) sequence to assess the recovery of motor units from fatigue. Five of these controls were randomly selected by the study team to return for a further two visits: once for reproducibility assessments of both PGSE‐MUMRI and PC‐MUMRI sequences, and a second time for dynamic serial 31P‐MRS to allow comparison of oxidative capacity with recovery of muscle contraction measures with the PC‐MUMRI paradigm (Figure 1).

### 2.3 Data Acquisition

#### 2.3.1 Imaging the Muscle Twitch Profile With PGSE‐MUMRI

**FIGURE 2:**
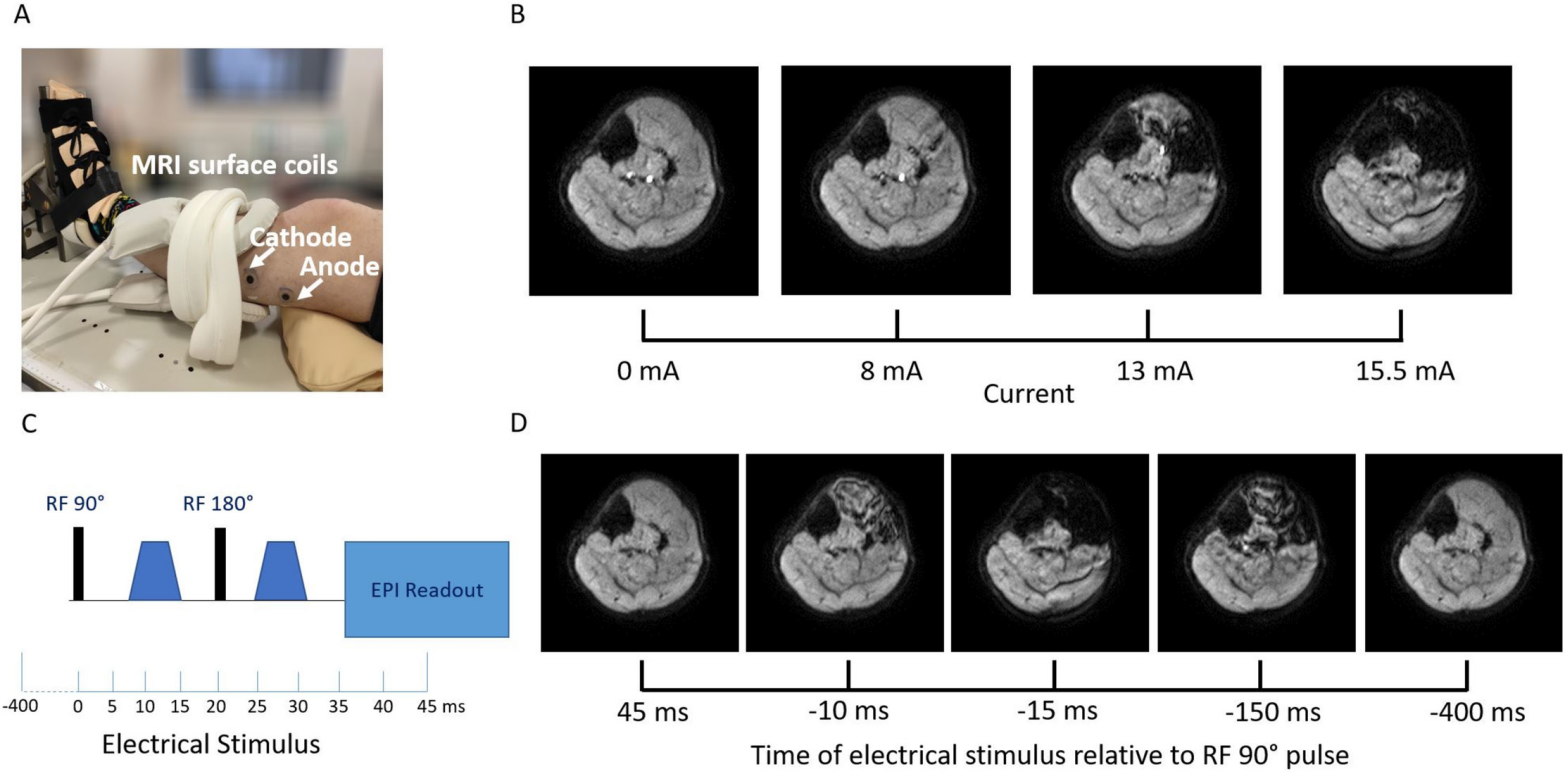
(A) Set‐up for a MUMRI experiment showing the placement of the surface coils and anode and cathode for common peroneal nerve stimulation. (B) Schematic of a pulsed gradient spin echo sequence (PGSE), representing altering of the electrical stimulus with respect to the 90° RF pulse, the stimulus was altered for each acquisition from 45 ms after the 90° RF pulse to 400 ms before the 90° RF pulse. (C) Example of a PGSE setup scan showing increasing stimulus current until maximum contrast between stimulated and non‐stimulated muscle is observed (at 15.5 mA). (D) Example images from a PGSE latency scan (twitch profile scan) showing images corresponding to different electrical stimulus delays relative to the RF 90° pulse.

The left foot was strapped into an MR compatible force plate, with stimulating electrodes placed over the common peroneal nerve and a pair of 10 cm flexible elliptical surface coils placed around the calf (Figure 2A) as previously described. First, the stimulation current (I_muscle_) defined as the current which gave maximal contrast between stimulated and unstimulated muscles was determined, by gradually increasing the current from 0 to ~20 mA, while acquiring a PGSE sequence with echo planar imaging read‐out (PGSE setup; Figure 2B; see Table 1 for imaging parameters).

**TABLE 1:** Relevant MRI parameters for all sequences used in the study.

Pulse sequence name	Field of view (mm)	Resolution (mm)	Slices	TR/TE (ms)	b value (s/mm^2^)	Δ/δ (ms)	Velocity encoding (VENC = cm/s)	Fat suppression	Number of dynamics	Number of averages	Acquisition time (mm:ss)
Pulsed gradient spin echo setup	160 × 60	1.5 × 1.5 × 8	2	1000/36	20	16.9/2.2	—	SPAIR, SSGR, olefinic fat	20	1	00:20
Pulsed gradient spin echo latency	160 × 160	1.5 × 1.5 × 8	2	1000/36	20	16.9/2.2	—	SPAIR, SSGR, olefinic fat	90	1	01:33
Bipolar gradient echo phase contrast latency	160 × 160	1.5 × 1.5 × 8	2	500/10	—	—	6	SPAIR, olefinic fat	90	1	03:01
Bipolar gradient echo phase contrast cyclic pre‐exercise	160 × 160	1.5 × 1.5 × 8	2	500/10	—	—	6	SPAIR, olefinic fat	90	1	03:01
Bipolar gradient echo phase contrast cyclic post‐exercise	160 × 160	1.5 × 1.5 × 8	2	500/10	—	—	6	SPAIR, olefinic fat	600	1	20:01
31P ISIS acquisition rest	—	—	—	25,000/0.1	—	—	—	—	—	4	01:04
31P ISIS acquisition exercise	—	—	—	5000/0.1	—	—	—	—	69	2	11:30

Abbreviations: Olefinic Fat – Custom pre‐pulse to null signal from olefinic fat; SPAIR – Spectral Adiabatic Inversion Recovery; SSGR – Slice Selective Gradient Reversal.

The twitch profile of the tibialis anterior (TA) muscle was then captured by repeating the PGSE sequence while stimulating the nerve with I_muscle_ with the stimulus gated between 400 ms before to 45 ms after the 90° radiofrequency pulse (PGSE latency; in steps of 5 ms, 90 acquisitions; Table 1 and Figure 2C,D) [16].

#### 2.3.2 Imaging Recovery From Muscle Fatigue With PC‐MUMRI

**FIGURE 3:**
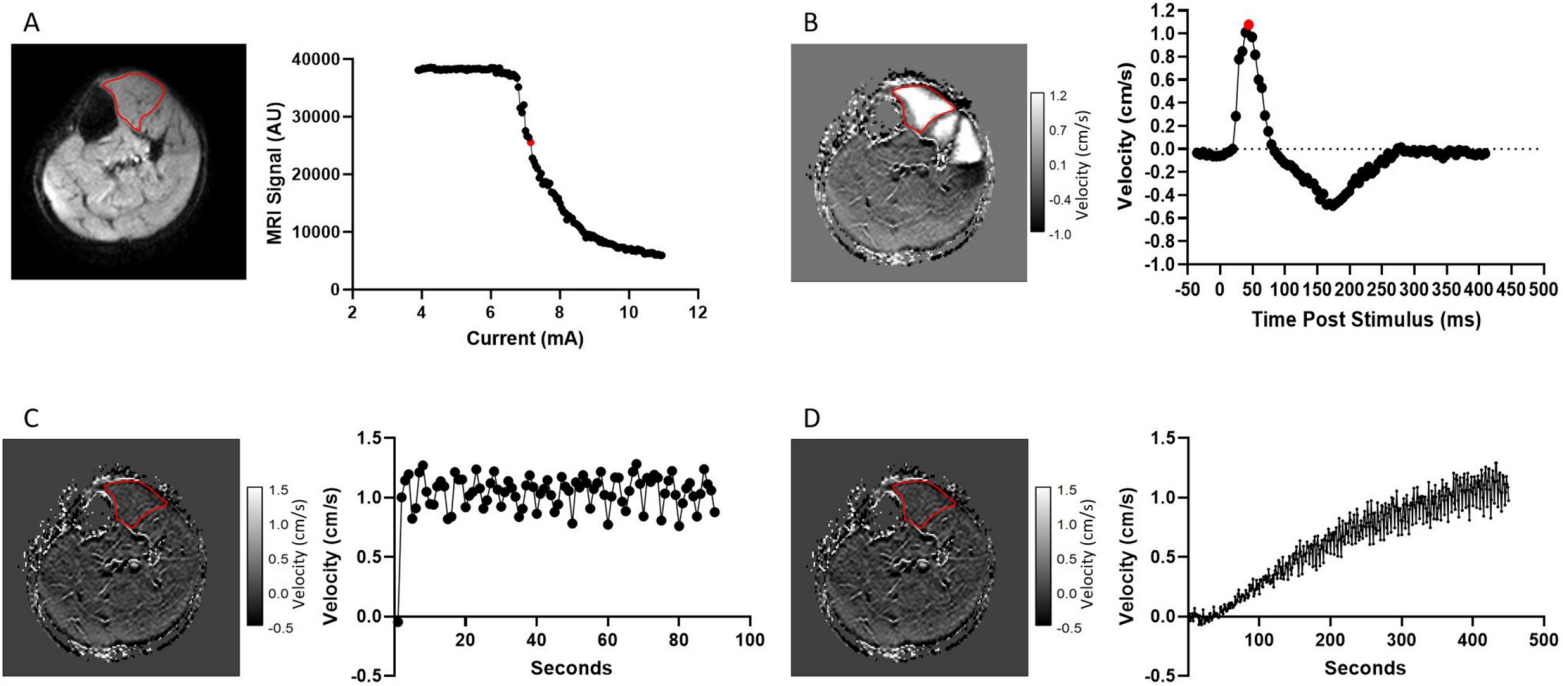
(A) A PGSE‐MUMRI sequence was used to identify the stimulation current for the subsequent phase contrast series to measure muscle fatigue. This was achieved by incrementally increasing the current through a series of MUMRI acquisitions—to standardise the measurement between subjects, the optimal current is defined at the value which results in a signal of 67% of the maximum (red point) average value within the TA (red outline). The TA muscle is delineated in red. (B) Bipolar gradient phase contrast acquisitions (VENC = 6 cm/s) are acquired at the current identified in panel A), with incrementally increased latency between stimulation and slice acquisition. This produces a muscle twitch velocity map, demonstrated here for the TA muscle. The peak velocity is easily identified (red point). (C) Pre‐exercise cyclic PC‐MUMRI. The expected latency of the peak velocity is sampled cyclically with four additional datapoints (−10 to +10 ms, step 5 ms) to account for any shift in peak velocity latency resulting from the muscle fatigue protocol. (D) Post‐exercise cyclic PC MUMRI. The subject performs fatiguing isometric exercise in the scanner, resulting in a loss of the stimulated motor unit twitch. Stimulated PC‐MUMRI scans were then collected using the cyclic sampling of the peak velocity for 20 min. The graph demonstrates a velocity near 0 cm/s directly following exercise that gradually recovers back to baseline values over time.

The maximum voluntary contraction force (MVC) was determined using the force plate by asking the participant to dorsiflex with maximum effort, with the highest of three attempts used as 100% MVC. For subsequent scans, in order to standardise the current across subjects, the current which results in a reduction of the PGSE signal to 67% of the unstimulated signal averaged across the TA was used as the stimulation current for each subject (Figure 3A). This stimulus level is comfortable enough for repetitive stimulation.

The participant then fully relaxed and the velocity twitch profile of the tibialis anterior (TA) muscle was captured using a bipolar gradient echo phase contrast sequence (PC latency), in a similar way to the PGSE twitch profile (detailed parameters in Table 1).

This obtained velocity twitch profile was used to identify the latency corresponding to the peak velocity of the TA muscle twitch (Figure 3B). To allow for any drift in this latency during the experiment we repeatedly sampled a 25 ms window in steps of five dynamics acquired with latencies −10, −5, 0, +5, +10 ms to the latency at which we expected the peak velocity to occur (Figure 3C) taking the highest value from each cycle as the peak velocity. We termed this cyclic PC‐MUMRI.

Muscle fatigue was achieved by asking the participants to perform sustained dorsiflexion at MVC for as long as possible. Participants were given visual feedback on their force production in real‐time, using a projector to display the force onto the scanner. Fatigue was defined once the maximum exerted force fell below 50% of the MVC. At this point, participants were instructed to relax, and the effect of the exercise and recovery were measured using the cyclic PC‐MUMRI over the next 1200 s (600 acquired dynamics resulting in 120 peak velocity measurements; detailed parameters in Table 1) (Figure 3D).

#### 2.3.3 Muscle Oxidative Capacity Measurements With 31P MRS

Five of the healthy controls were asked to return on a different day to measure the response of the muscle to fatigue using 31P‐MRS. This was performed in the same leg and slice location as the MUMRI measurements. A 14 cm diameter phosphorus surface transmit/receive coil was attached to the widest anterior part of the muscle of the lower leg. Phosphorus spectra were acquired using a 1D ISIS sequence to limit the signal acquisition to the anterior muscle compartment (predominantly TA). Fully relaxed spectra were acquired from the anterior compartment at rest (parameters in Table 1). For the dynamic exercise challenge, the same MVC based fatigue protocol was used as for the PC‐MUMRI test. After the exercise, participants were asked to remain as still as possible, spectra were acquired immediately after the exercise stopped and were recorded during recovery for at least 1200 s.

### 2.4 Part II: Interventional Study in Patients With PMM

To evaluate the use of the MUMRI fatigue protocol to assess differences between health and disease and response to intervention, we conducted an interventional study in patients with PMM.

#### 2.4.1 Patients

Nine patients with single large‐scale mtDNA deletion PMM (mean ± SD, age: 59.6 ± 10.7) were recruited. Ethical approval for the single‐group interventional study in patients with PMM was obtained from an NHS REC (HRA and Health and Care Research Wales, Project Reference: 21/NW/0316, Ethical Approval Number: 304444). In accordance with this approval, potentially eligible patients with PMM were identified by the Newcastle Clinic for Research Themed Assessment (CRESTA) mitochondrial group. They were subsequently screened by a clinician to ensure that an exercise intervention would be safe, given the high incidence of cardiovascular morbidity in patients with PMM. Written informed consent was obtained before the patient attended for the study visit. Patients varied in phenotype with some patients being more active than others.

#### 2.4.2 Exercise Programme and Clinical Review

All subjects underwent baseline PGSE‐MUMRI and PC‐MUMRI assessments as described in Part I, and a clinical review performed by a suitably trained member of the mitochondrial research team. This included manual muscle testing, grip strength (measured using a Jamar Dynamometer; Lafayette Instruments, Lafayette, Indiana, USA), timed single leg stance and 30 s sit to stand.

Patients then undertook a 12‐week home‐based exercise programme (Giraffe Healthcare [20]) with fortnightly remote monitoring. Patients were prescribed two exercise sessions per week (total target: 24 sessions), with at least 24 h rest between sessions. Each session consisted of a 5‐min aerobic warm up, structured exercises, and a cool down with static stretches. Each participant completed 7–10 exercises per session, predominantly composing lower limb strength and balance, including mandatory exercises to activate the TA. Each exercise was tailored according to the ability of the patient, with resistance applied via body weight, a resistance band, or adjustable ankle weight (Sportneer, UK) as appropriate. Patients recorded completed exercises and reported perceived symptoms of fatigue and pain in a weekly diary. Individual progress was reviewed through fortnightly telephone calls, with exercises progressively increased if suitable (based on the judgement of the research team). For those patients who completed the exercise programme, a measure of compliance was taken as the percentage of exercises recorded as fully completed over the 12‐week period.

Following completion of the exercise programme, patients were invited for a follow‐up clinical review and PGSE‐MUMRI and PC‐MUMRI assessments. 31P‐MRS measurements were not made in the PMM study group, as it was felt too onerous to ask the patients to return for a third visit and oxidative capacity measurements in PMM were not the aim of this study.

### 2.5 Data Analysis of Part I and Part II

#### 2.5.1 Muscle Twitch Profile With PGSE‐MUMRI

**FIGURE 4:**
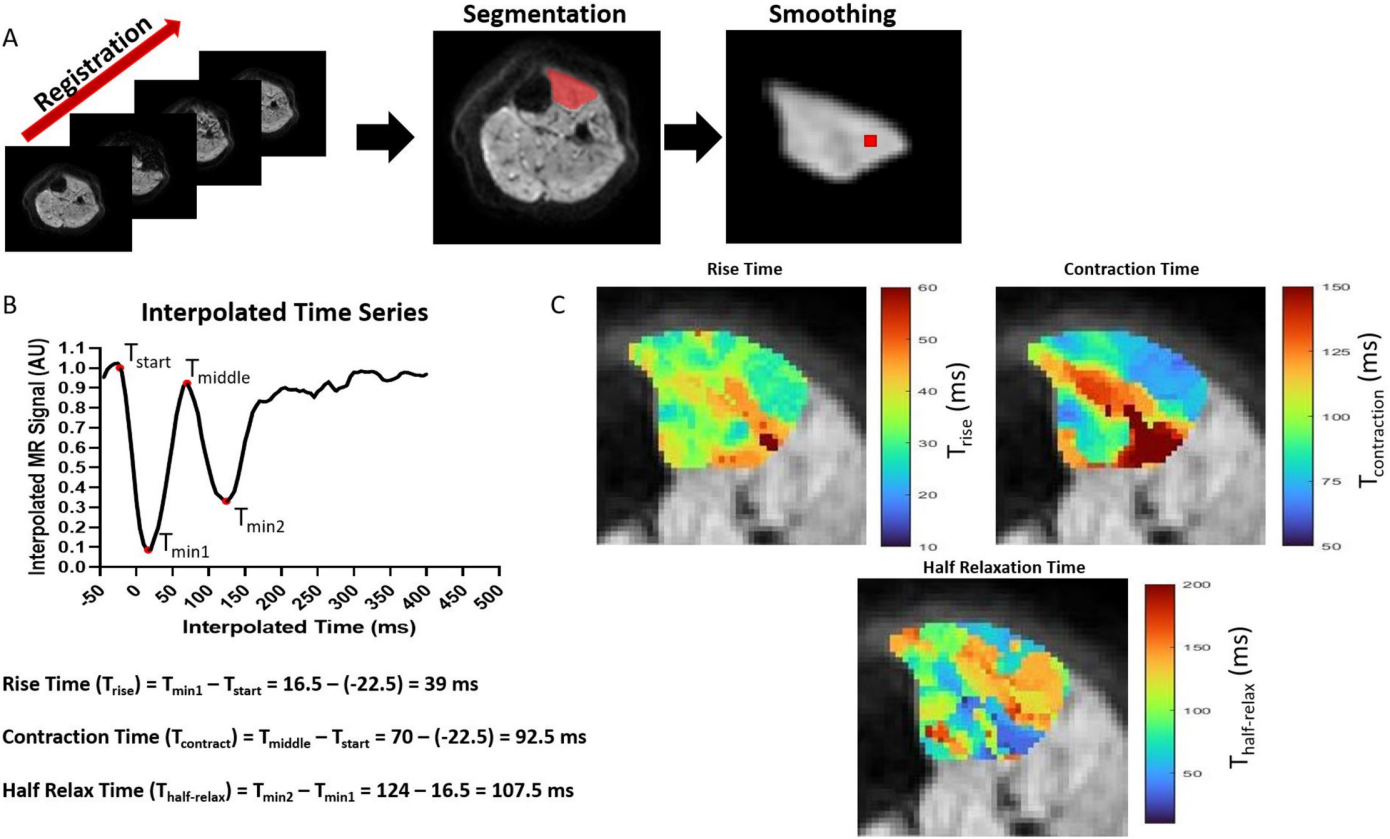
(A) Data processing steps. First registration of the time‐series to the first image. Secondly, segmentation of the TA muscle. Finally, smoothing of the segmented image and interpolation of the time series. (B) Graph shows smoothed and interpolated signal from the red voxel in the smoothed image. The red points show the automatically extracted times to calculate rise time (T_rise_), contraction time (T_contract_) and half relaxation time (T_half‐relax_). Example calculations are shown underneath for the red voxel. (C) This process is repeated for all voxels within the region of interest to create parametric maps. Each map has its own scale.

Images were registered to the first image within the time series using a nonrigid registration in Matlab version 9.10.0 R2021a (Natick, Massachusetts: The MathWorks Inc.; 2021). The TA was manually segmented in ITKSnap (version 3.8.0) [21] and analysis of segmented voxels within the TA was performed in Matlab. The time‐series data from each voxel were extracted, smoothed using a moving average and interpolated using a linear function to an effective time step of 0.5 ms (Figure 4A).

For each time‐series four points were automatically calculated (Figure 4B): the beginning of the signal change (T_start_), defined as the first time point at which the signal decreased to a value three times less than the standard deviation of the baseline; the first signal minimum (T_min1_); the point closest to the baseline between the first and second signal minima (T_middle_); and the second signal minimum (T_min2_), see Figure 4B. Voxel‐wise twitch rise time (T_rise_ = T_min1_ − T_start_), contraction time (T_contract_ = T_middle_ − T_start_) and half‐relaxation time (T_half‐relax_ = T_min2_ − T_min1_) of the TA were then calculated and the average of all voxels taken. Figure 4C shows three example parametric maps for T_rise_, T_contract_ and T_half‐relax_.

In order to further investigate the change in rise time, contraction time and half‐relaxation time following the 12‐week resistance exercise intervention, histogram analysis was performed. Pooled across all PMM patients, the frequency of voxels with a given rise time, contraction time and half relaxation time were represented as histograms using 50 bins per histogram. A nonparametric kernel smoothing distribution was fitted to each histogram to represent the distribution. Finally, for each participant, thresholded parametric maps for the MUMRI metrics were produced, these were created by categorising each voxel in the mask according to the individual histogram values into four‐time groups of equal spacing across the range of the metric.

#### 2.5.2 Recovery From Muscle Fatigue With PC‐MUMRI

As for the PGSE‐MUMRI images, the phase contrast post‐fatigue recovery data was registered to the first image within the time series using a nonrigid registration in Matlab. FIJI [22] was used for manual segmentation of the TA. A custom‐written Matlab script was used to extract the peak velocity across the five latencies in each cycle for each point in the recovery period. The time course of the recovery of the muscle twitch in the resulting data was modelled empirically as a modified Gompertz recovery curve using Equation (1) from which the time‐to‐half‐maximum (T_half‐max_) of TA peak velocity recovery were calculated.

(1) $$y=a\exp^{-b\exp^{-\mathrm{cx}}}-d$$

Where a is an asymptote, b controls translation of the recovery curve, c is a scaling factor for the growth rate, x is the time variable, and d is an extra offset term.

#### 2.5.3 Muscle Oxidative Capacity With 31P MRS

Phosphorous spectra were analysed by time‐domain fitting using the AMARES algorithm in jMRUI (version 5.2) [23]. Spectral peaks from phosphocreatine (PCr) were modelled using Lorentzian line shapes with a fixed zero order phasing.

The post‐exercise phosphocreatine areas were fitted to a mono‐exponential curve to calculate the recovery coefficient kPCr.

#### 2.5.4 Statistics

Statistical analysis was performed with GraphPad Prism (version 10.0.0 for Windows, GraphPad Software, Boston, Massachusetts, USA). Repeatability was assessed for each variable using the absolute average percentage difference between the two scans. PCr recovery times measured using 31P‐MRS were compared to T_half‐max_ measured using PC‐MUMRI using a paired parametric Student's *t*‐test.

T_rise_, T_contract_ and T_half‐relax_ were compared between controls and PMM patients at baseline using unpaired parametric Student's *t*‐tests. T_rise_, T_contract_, T_half‐relax_ and T_half‐max_ were compared as individual metrics for PMM patients between baseline and following the 12‐week exercise programme using paired parametric Student's *t*‐tests. Results are presented as mean ± standard deviation and statistical significance was set at *p* < 0.05.

## 3 Results

### 3.1 Repeatability and Validity

**FIGURE 5:**
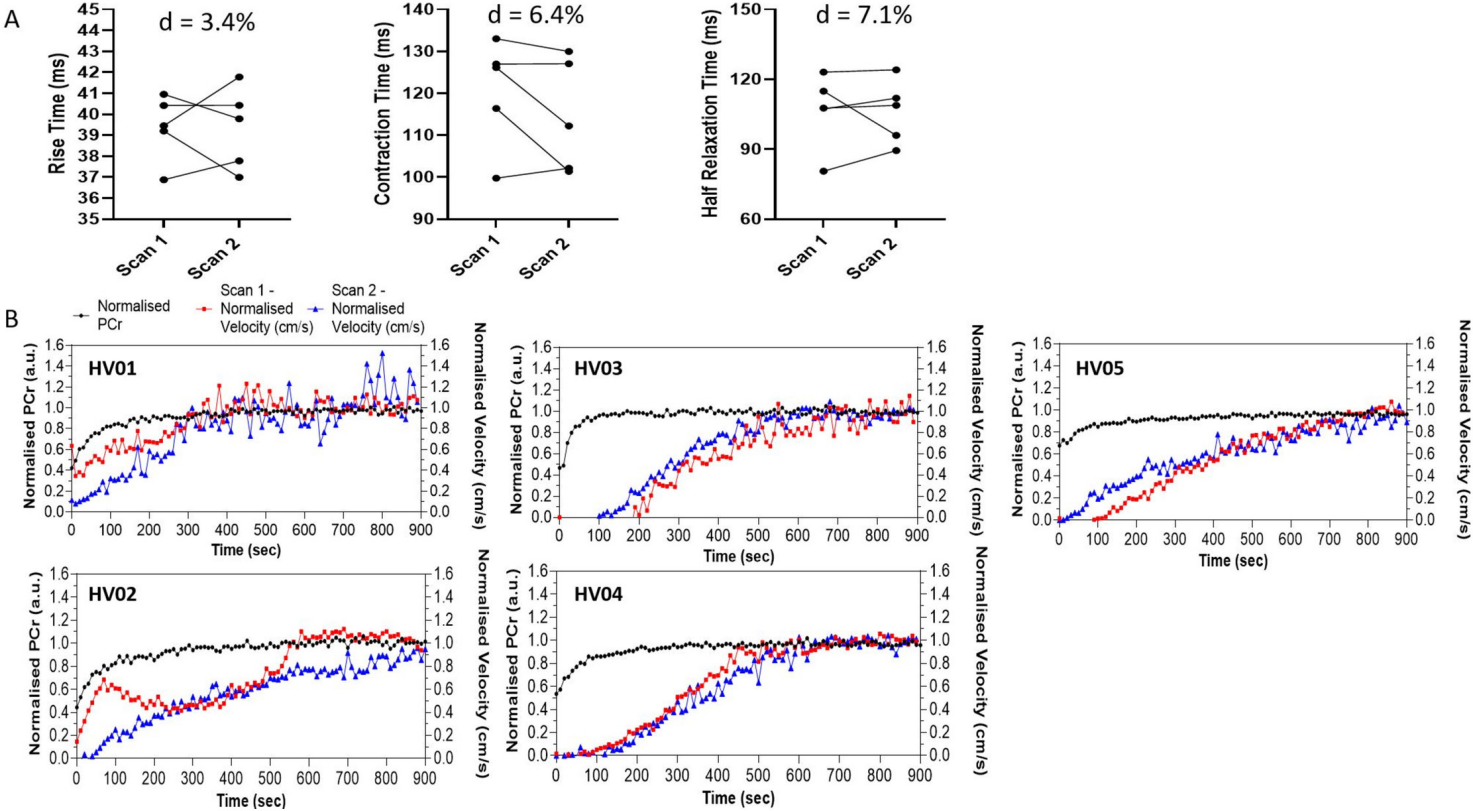
(A) Reproducibility plots for rise time, contraction time, and half relaxation time measured with PGSE‐MUMRI for five controls, d is the mean percentage difference in the metric between scan 1 and 2. (B) Post‐fatigue twitch velocity recovery curves measured with PC‐MUMRI of five healthy volunteers, collected in separate sessions. Data are normalised to end recovery velocity—red (scan 1) and blue (scan 2). Corresponding 31P recovery curves are plotted in black.

Five healthy controls underwent two assessments (mean time between assessment: 29 ± 21 days). The absolute percentage difference for the muscle twitch parameters measured with PGSE‐MUMRI for the two scan sessions were 3.4% for T_rise_, 6.4% for T_contract_ and 7.1% for T_half‐relax_ (Figure 5A). Phase contrast derived twitch recovery curves appeared reproducible, with no significant difference in time to T_half‐max_ between scans (scan 1:238 ± 156 vs. scan 2:314 ± 31 ms, *p* = 0.263), and demonstrated a consistently longer recovery time constant than those acquired from 31P‐MRS (Figure 5B). Across the five subjects, end‐exercise depletion of PCr was 50 ± 10% and half time for PCr recovery was 65 ± 27 s. It is notable that the PCr recovery time is significantly shorter than the average muscle twitch recovery time in the same five healthy controls T_half‐max_ = 346 ± 97 s (*p* = 0.002).

### 3.2 Interventional Study in PMM Patients

#### 3.2.1 Comparison of Twitch Characteristics in Healthy Volunteers and PMM Patients at Baseline

**FIGURE 6:**
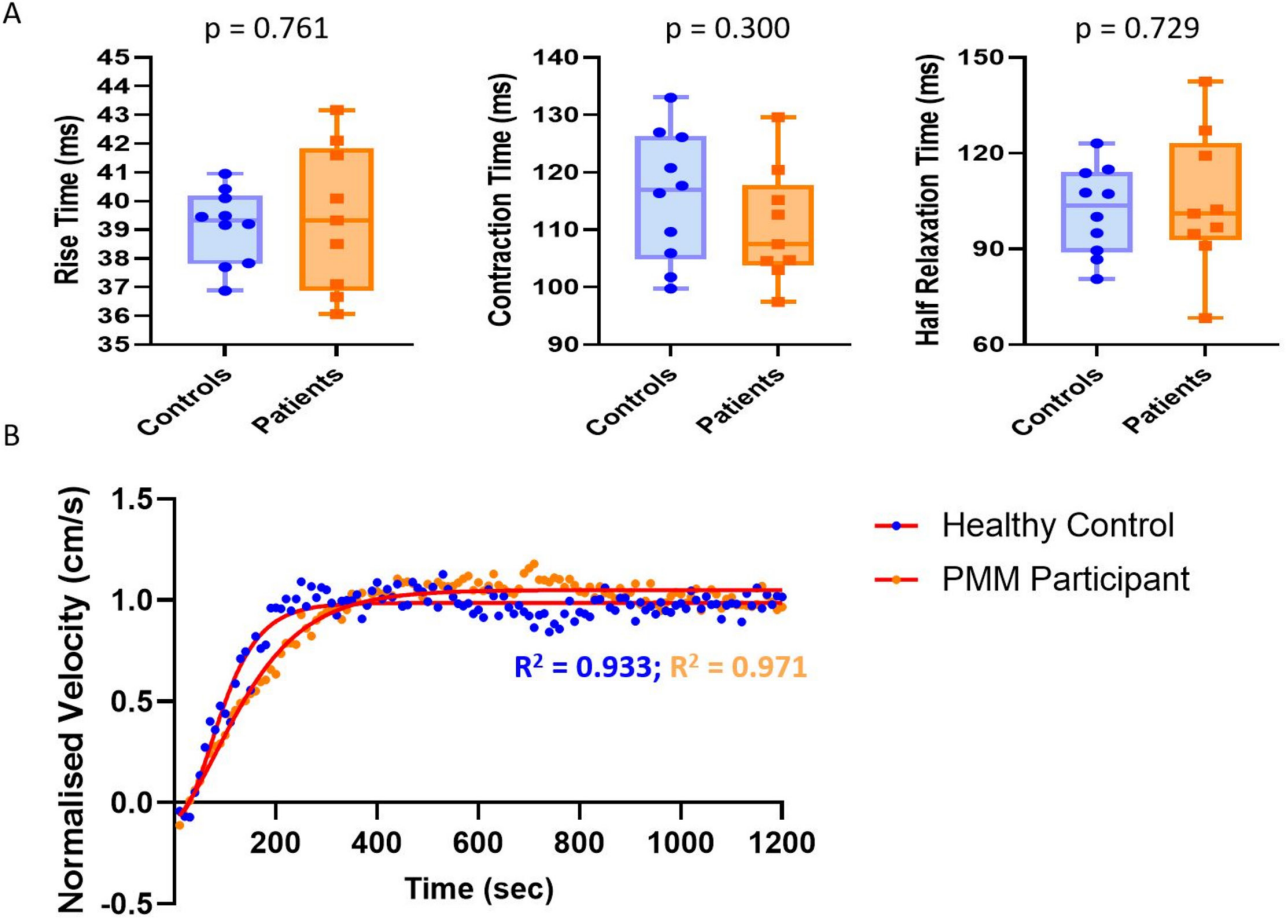
(A) Box plots of rise time, contraction time and half relaxation time comparing healthy controls (blue) and patients with primary mitochondrial myopathy (PMM, orange) at baseline. (B) Two example PC‐MUMRI recovery curves from a healthy control (blue) and PMM patient (orange) fitted with the five‐parameter Gompertz model, *R* ^2^ values are shown on the graph.

At rest, no significant differences in T_rise_, T_contract_ and T_half‐relax_ were detected between healthy controls and PMM patients (T_rise_: 39.1 ± 1.3 vs. 39.4 ± 2.5 ms; *p* = 0.761, T_contract_: 115.8 ± 11.3 vs. 110.6 ± 10.0 ms; *p* = 0.300, and for T_half‐relax_: 101.9 ± 13.8 vs. 104.8 ± 21.9 ms; *p* = 0.729) (Figure 6A). Following fatiguing exercise, the five‐parameter Gompertz model used to fit the PC‐MUMRI recovery data demonstrated an adjusted R‐squared at group level of 0.94. T_half‐max_ did not differ between controls and PMM patients (262 ± 103 vs. 247 ± 98 s; *p* = 0.779) (Figure 6B).

#### 3.2.2 Response to Structured Exercise Programme in PMM Patients

Of the nine PMM patients who underwent the baseline assessment, two patients were lost to follow up. One patient was diagnosed with cancer unrelated to their PMM, and one found the travel to the imaging centre too onerous. Of the remaining seven patients who completed the exercise programme, compliance with the exercise programme was: 82.5% (one patient), 93% (one patient) and 100% (five patients). Two follow‐up scans were excluded (one because of inadequate stimulation of the tibialis anterior muscle, and one because of signal dropouts in the data), resulting in seven pre– and post–PGSE‐MUMRI data sets and five pre‐ and post‐exercise PC data sets.

**FIGURE 7:**
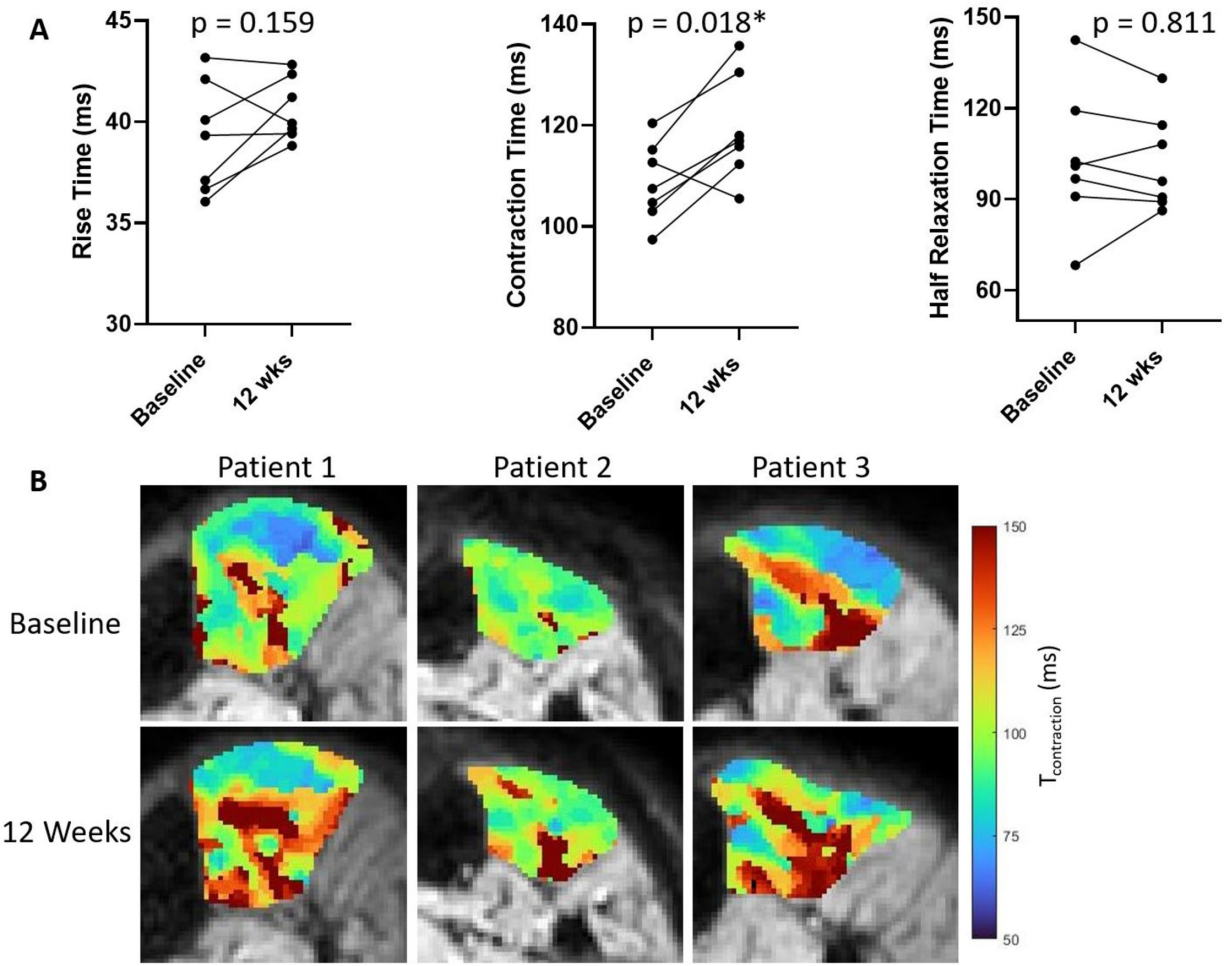
(A) Dot plots for rise time, contraction time, and half relaxation time for patients with primary mitochondrial myopathy pre and post 12‐week exercise programme. This demonstrates a significantly longer contraction time post‐exercise programme. (B) Example parametric maps for contraction time from three patients, top row is baseline and bottom row is post 12‐week exercise programme, demonstrating regional changes in contraction time.

Analysis of the twitch characteristics shows that T_contract_ was significantly longer after the structured exercise programme (pre 108.7 ± 7.9 vs. post‐119.3 ± 10.4 ms; *p* = 0.018). There was no difference in T_rise_: 39.2 ± 2.8 vs. 40.6 ± 1.5 ms, *p* = 0.159; or T_half‐relax_: 103.0 ± 23.1 vs. 102.1 ± 16.0 ms, *p* = 0.811 (Figure 7A,B).

**FIGURE 8:**
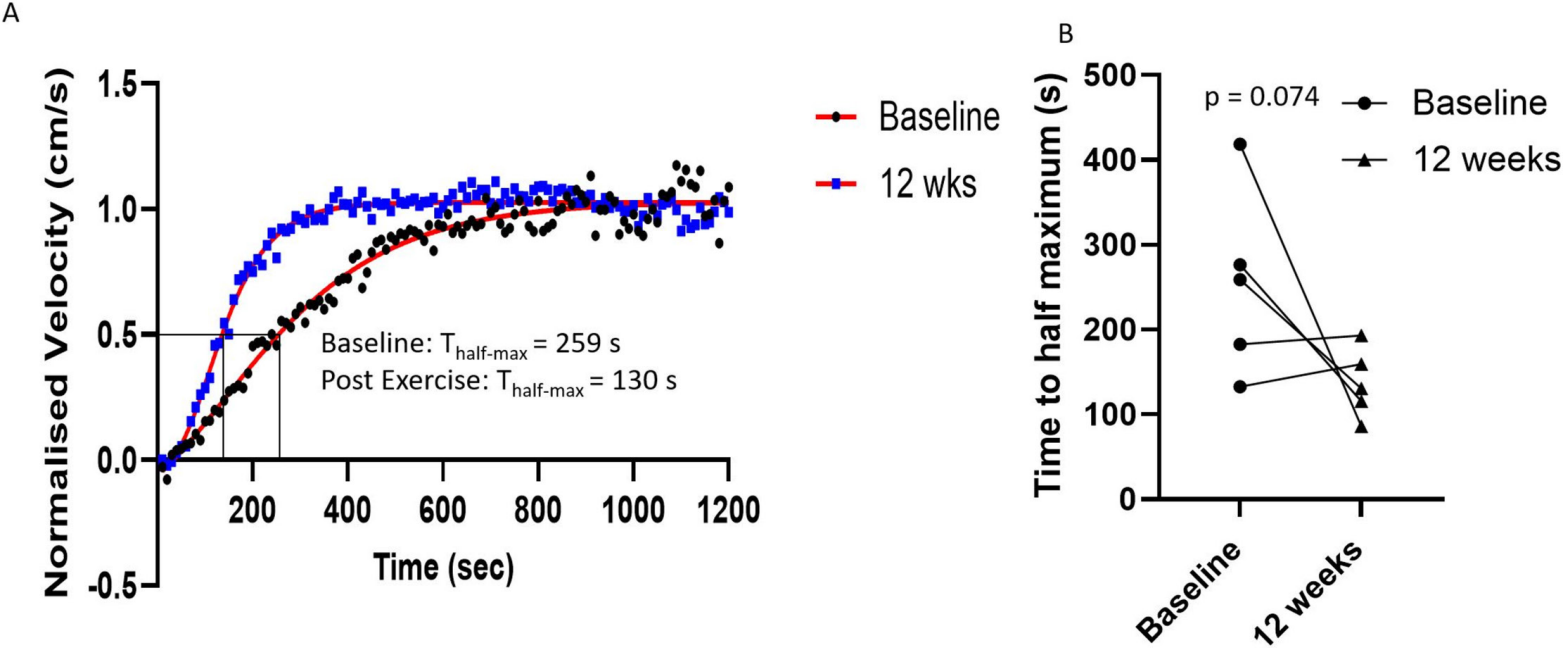
(A) Example twitch velocity post‐fatigue recovery curves in a participant with PMM, before (black dots) and after the 12‐week exercise programme (blue squares). Velocity data plotted with fitted model (red lines) and the time to half‐maximum (T_half‐max_). (B) T_half‐max_ of the phase contrast velocity recovery curves before (circles) and after (triangles) a 12‐week exercise programme in five patients with PMM. The three patients with the highest initial T_half‐max_ demonstrate changes consistent with faster twitch recovery following the exercise programme. The remaining two patients did not demonstrate a significant change in the twitch recovery—we note that these had the quickest initial T_half‐max_.

Muscle fatigue assessments with T_half‐max_ demonstrated a trend towards quicker recovery from fatigue post‐exercise programme, though this did not reach the threshold for significance: 254 ± 109 vs. 137 ± 41 s; *p* = 0.074. Figure 8A shows two example curves in one patient at baseline and post‐exercise programme. The reduction in T_half‐max_ was predominantly observed in the three patients with the longest values at baseline (Figure 8B).

#### 3.2.3 Comparison of Twitch Characteristics and Clinical Review Data

Correlations between manual muscle testing, grip strength, single leg stance and sit to stand test with muscle twitch contraction time measured using PGSE‐MUMRI are shown in Table S1. Following Bonferroni correction for the number of tests performed, there were no statistically significant correlations observed.

#### 3.2.4 Regional Differences in Twitch Dynamics

**FIGURE 9:**
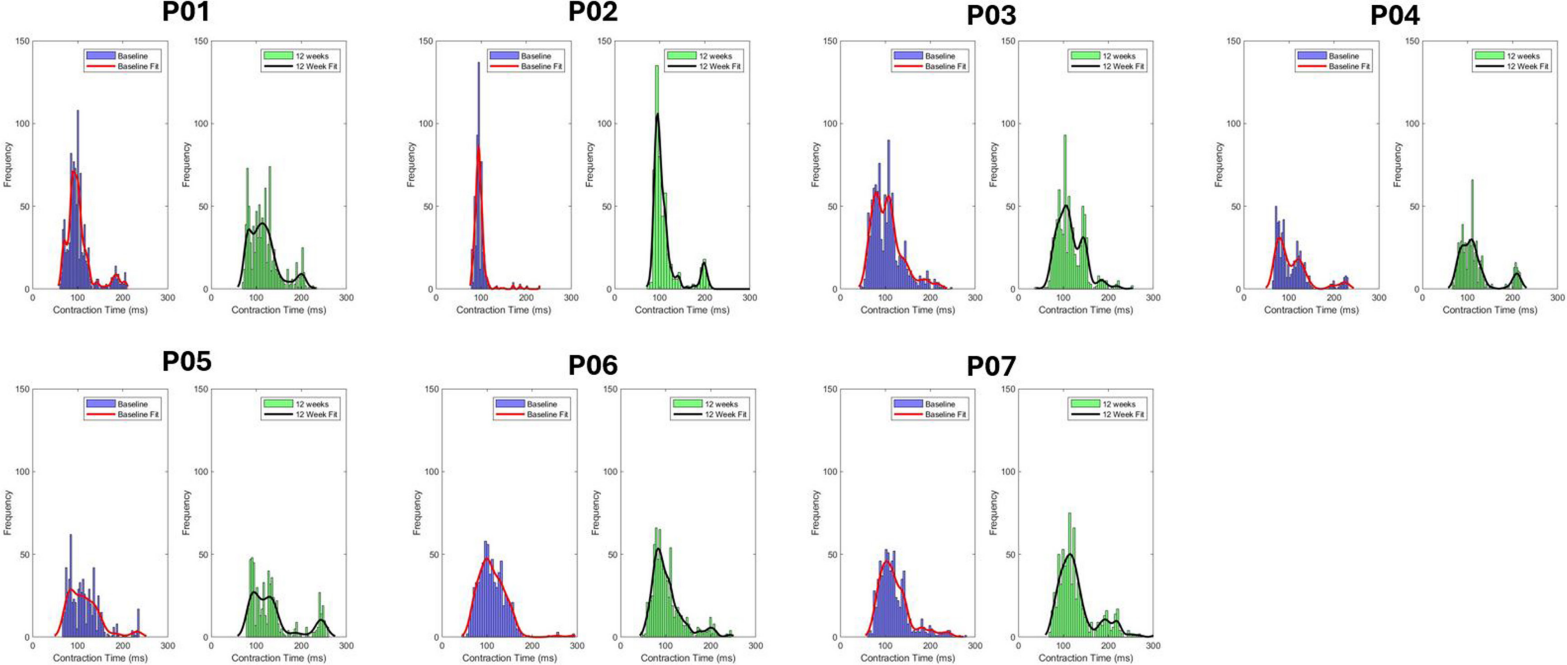
Histograms of the contraction time for each patient (P01–P07) on a voxel‐wise basis at baseline (blue bars; left) and at 12 weeks post‐exercise programme (green bars; right). A nonparametric fit is applied to show distribution (baseline—red; 12 weeks—black).

Having demonstrated a significant difference in contraction time over the whole TA muscle, we next compared the pre‐and post‐exercise programme scans on a voxel‐by‐voxel basis. Figure 9 shows histograms of contraction time for all voxels in each of the seven PMM patients between baseline and follow up. Histograms of contraction time at baseline for control subjects are shown in Figure S1. The baseline histogram for contraction time showed a predominant peak around 100 ms with two further peaks at longer contraction times (> 200 ms). Post‐exercise, these two peaks were much more obvious, particularly with a contraction time around 200 ms.

**FIGURE 10:**
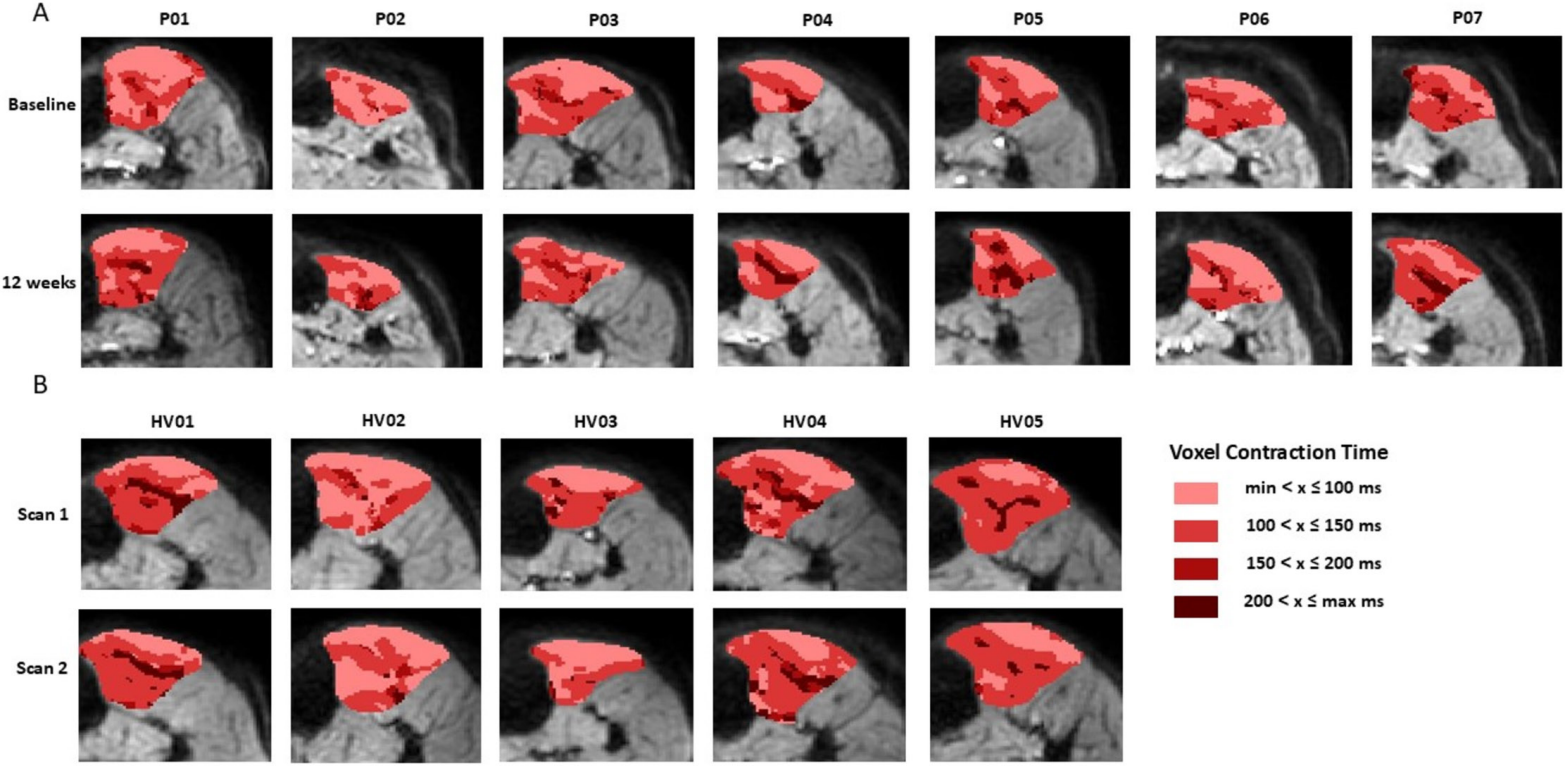
(A) Thresholded voxel wise maps of contraction time for each participant at baseline (top row) and at 12 weeks (bottom row). (B) Thresholded voxel wise maps of contraction time for the five healthy controls who underwent two scans scan 1 (top row), scan 2 (bottom row). Thresholds applied are based on the histogram distributions: light pink represents contraction times greater than the minimum value from the histogram and less than or equal to 100 ms; red represents contraction times greater than 100 and less than or equal to 150 ms; dark red represents contraction times greater than 150 ms and less than or equal to 200 ms; maroon represents contraction times greater than 200 ms and less than or equal to the maximum value from the histogram.

We next examined the distribution of twitch times within the TA muscle in each individual patient. In the baseline scans there were clear regional differences in the twitch dynamics, with superficial regions of the muscle showing a shorter contraction time than deeper regions (Figure 10A). The tibialis anterior is a pennate muscle with fibres inserting into a central aponeurosis, fibres in the area surrounding this aponeurosis showed the longest contraction times shown by the dark regions in Figure 10A. For comparison with healthy controls maps for the five controls who underwent two scans assessing for reproducibility are shown in Figure 10B.

## 4 Discussion

Our aim was to develop a reproducible method of studying dynamic changes in muscle function suitable for use in longitudinal patient studies, and capable of providing greater spatial information than 31P MRS. As a proof‐of‐concept we achieved this; the scan protocol was well tolerated. Two patients dropped out of the study, although they did so for unrelated reasons. The PGSE protocol produced reproducible results in the healthy controls, with the average change in measured MUMRI metrics (rise, contraction and half‐relaxation time) being 5.6%, which is factor of two lower than the changes we observed in PMM patients after a 12‐week exercise intervention (~10%). The in‐scanner PC‐MUMRI fatiguing protocol proved more challenging to perform, with incomplete data sets in two of the control subjects and two of the patients, either because of unexplained signal drop‐out or inadequate nerve stimulation.

Muscle fibres are categorized based on their contractile properties and resistance to fatigue [24]. The contractile properties of the muscle fibre are closely related to the kinetics of calcium release from and re‐uptake to the sarcoplasmic reticulum, the instantaneous force produced reflecting a low pass filtered calcium signal [25]. We used two versions of our MUMRI technique in order to quantify both these features. First; PGSE is highly sensitive to actively contracting muscle fibres, but not to the resulting passive movement of surrounding tissues [16]. For this reason, it provides a high‐resolution image of regional muscle fibre activity which we have previously used to determine the size and shape of individual motor units using time‐locked motor nerve stimulation [26]. We further developed this technique by systematically varying the latency between motor nerve stimulation and the PGSE image in order to capture the twitch dynamics of the entire TA muscle at single voxel resolution (approximately 1.5 × 1.5 mm). The pooled data from all the PMM patients showed a bimodal distribution of contraction times, the majority of fibres having contraction times around 100 ms, with a second peak at around 200 ms. These times agree with previously reported motor unit twitch times in the lower limb ranging between 38 and 251 ms [27, 28].

A unique feature of the PGSE‐MUMRI technique is that it provides an indication of the distribution of twitch dynamics across the entire muscle. At the individual patient level, we were able to demonstrate a highly variable spatial distribution of these twitch profiles albeit with clear regional differences. Muscle fibres with the fastest contracting fibres predominantly occupied the superficial regions of the muscle, with the slowest contracting fibres predominantly occupying deeper regions adjacent to the central aponeurosis.

Muscle fibre type can shift in response to exercise [29] with endurance training resulting in a shift towards type 1 slow twitch fibres. Mitochondria are abundant in type I fibres in which oxidative metabolism predominates. We were interested to see whether the PGSE protocol was sufficiently sensitive to detect these changes in response to a 12‐week home exercise programme. This was designed to provide daily endurance training for the TA muscle, and we achieved a high rate of compliance through regular on‐line follow‐up. Following this, we demonstrated a significant increase in contraction time which may be consistent with an increase in the activity of type 1 fatigue‐resistant fibres. This is in line with previous studies of endurance training in mitochondrial disease, which showed an increase in aerobic exercise capacity and mitochondrial DNA mass [30, 31, 32].

Comparing the voxel‐wise histograms before and after exercise suggested that rather than there being a gradual shift in all of the fibres towards a slow‐twitch phenotype, there was a marked increase in the activity of the slowest contracting fibres with contraction times around 200 ms. The distribution of these fibres was not random but rather occurred in the central region of the muscle, around the central aponeurosis. Post exercise, the area occupied by fibres with the shortest contraction times (i.e., those between the minimum value of the histogram and less than or equal to 100 ms) was reduced in six out of the seven subjects. In these subjects, there was also an increase in the area occupied by fibres with the longest contraction times, particularly around the central aponeurosis (see e.g. P04 and P07). The exception was P06, in whom no clear change in fibre contraction time distribution was seen.

Unsurprisingly, there is very limited data on regional differences in muscle fibre properties in human muscles. Henriksson‐Larsen et al. obtained sections of the entire TA muscle from six previously healthy males who had suffered a sudden accidental death and showed a highly variable distribution of muscle fibre types based on mATPase staining, albeit with a possible increase in type 1 fibres in deeper regions [33].

It takes approximately 1.5 min to acquire a twitch profile using the PGSE‐MUMRI technique above. This makes it unsuited to investigating fatigue, in which muscle fibre properties vary over a time course of several seconds. For this, we used a variation of our previously published PC‐MUMRI sequence. This has the advantage of providing a rapid measure of muscle twitch velocity over time but has a lower spatial resolution than the PGSE sequence. This is because it is sensitive not only to the actively contracting motor unit but also to the surrounding muscle tissue that this motor unit passively distorts [16].

We developed a novel muscle twitch peak velocity fatigue‐recovery paradigm which produced consistent recovery curves which were fitted to a model to extract the time‐to‐half‐maximum T_half‐max_. Our fatiguing protocol produced profound decreases in peak velocity, which recovered with a time course markedly longer than the corresponding 31P MRS recovery curves. We suspect that this is because whereas the 31P MRS is sensitive to upstream changes in mitochondrial function and ATP production, the PC‐MUMRI sequence reflects changes in excitation‐contraction coupling which are several stages down‐stream of these, with each stage having its own recovery profile. For example, calcium release and uptake by the endoplasmic reticulum can remain depressed for several minutes following strenuous exercise [34]. Hence, 31P MRS and PC‐MUMRI may provide complimentary information on the energetics of muscle contraction.

The PC‐MUMRI could not be successfully acquired in two healthy controls and two patients. No statistically significant differences between controls and patients were observed at baseline or in the patient group following the exercise programme. Despite the small sample size, the PC‐MUMRI T_half‐max_ reveals a trend towards faster recovery from fatigue. This is consistent with the increase in twitch contraction time seen in PGSE data and may be suggestive of improved mitochondrial function. However, this would require confirmation in any follow up studies.

There were a number of limitations to the study. Firstly, measurements of muscle twitch dynamics made with the PGSE sequence were only in a single slice along the length of the muscle and was limited to assessment of the TA. It would be of interest to study how muscle twitch dynamics vary along the length of muscles and between different muscle groups. This would be particularly relevant in studies of neuromuscular diseases which appear to selectively affect muscles. For example in sarcopenia the quadriceps muscles appear to lose muscle mass and strength before other lower limb muscles [35]. Secondly, the study is limited by the lack of a muscle biopsy to verify if the changes observed in twitch dynamics post exercise programme are indeed due to the proposed changes to muscle fibre type. This study was designed as a pilot study, and we did not feel it was ethically justified to subject patients to repeated muscle biopsy before we had shown that the MUMRI technique was able to detect changes pre‐ and post‐exercise. In future, it will be important to validate the observed changes in muscle twitch dynamics with muscle biopsy. However, as Henriksson‐Larsen et al. cautioned, the striking regional differences in fibre typing seen in their post‐mortem specimens, and supported by our regional contraction maps, could result in misleading results from muscle biopsies which are in effect a small sample of fibres from a random location within the muscle [36]. Although all of the patients included in Part II of the study were single large‐scale mtDNA deletion, they varied in phenotype; this will have introduced some bias to the results; however, these patients were difficult to recruit and so for proof‐of‐concept it was felt that a wide range of phenotypes would not adversely affect the aims of the study. We did not collect physical activity data in healthy volunteers or participants at baseline, this was due to limited resources. As this is an important confounding factor measurements of activity levels should be included in any follow‐on studies. Finally, we did not perform the exercise intervention in the healthy volunteers, this was primarily due to the capacity and budget to deliver this; however, this would be an important inclusion in any follow‐on studies.

## 5 Conclusions

We have further developed the MUMRI technique to study the dynamic function of muscle including novel measurements of muscle twitch dynamics and fatigue. The technique is well tolerated, and at least for the muscle twitch dynamics assessment with the PGSE sequence appears reproducible and sensitive to exercise‐induced changes in muscle fibre properties. Uniquely, it provides an in vivo image of fibre twitch dynamics within human muscle. This pilot study does require further validation particularly with muscle biopsy, but nevertheless, we feel that it shows potential as an entirely non‐invasive trial biomarker for future studies of exercise‐induced muscle adaptations.

## Conflicts of Interest

The authors declare no conflicts of interest.

## Supporting information


**Table S1.** Shows *R* ^2^ and *p* value from simple linear correlation between measured contraction time (ms) and the physical performance test metric. Statistical significance set at (*p* < 0.0056) following Bonferroni correction.
**Figure S1.** Histograms of contraction time for each healthy volunteer at baseline on a voxel wise basis. A nonparametric fit is applied to show distribution (red line).

## Data Availability

The data that support the findings of this study are available from the corresponding author upon reasonable request.
