# Supplementary material for: Measurement of Twitch Dynamics in Response to Exercise Induced Changes in Mitochondrial Disease Using Motor Unit Magnetic Resonance Imaging (MUMRI): A Proof‐of‐Concept Study
Source: NMR Biomed. 2025 Apr 9;38(5):e70021. doi: 10.1002/nbm.70021 (PMC11981886; doi:10.1002/nbm.70021)
Supplement: Supplementary file 1 — Table S1. Shows R 2 and p value from simple linear correlation between measured contraction time (ms) and the physical performance test metric. Statistical significance set at (p < 0.0056) following Bonferroni correction. Figure S1. Histograms of contraction time for each healthy volunteer at baseline on a voxel wise basis. A nonparametric fit is applied to show distribution (red line). [file NBM-38-e70021-s001.docx]

**Supplementary Materials**

Inclusion and exclusion criteria

Healthy controls

- Willing and able to provide informed consent
- Age >= 16

The exclusion criteria were:

- Contra-indication to MRI
- Inability to perform a dorsiflexion fatiguing protocol
- Any chronic medical condition or acute injury which could affect the muscles.

Patients with PMM

The inclusion criteria were:

- Willing and able to provide informed consent
- Adults >= 16 years old at the time of recruitment
- Genetically confirmed diagnosis of PMM (specifically, single mitochondrial deletion)
- Lower limb weakness, in the opinion of the recruiting investigator
- Ability to undergo all trial assessments, in the opinion of the recruiting investigator
- Willing and capable to engage with all intervention requirements
- Able to perform active ankle dorsiflexion and plantarflexion actively against gravity

The exclusion criteria were:

- Contra-indication to MRI (eg., implanted cardiac defibrillator or cochlear implant)
- Inability to achieve actively plantarflex the ankle
- Uncontrolled cardiovascular or metabolic disease
- Resting hypertension (>= 180/100 mmHg) or tachycardia (>= 100bpm)
- Breathlessness at rest or with mild exertion
- Inability to understand written and verbal instructions in English
- Physical disability or cognitive impairment that precluded safe and adequate participation in the trial
- Evidence of clinically significant heart disease based on the clinical judgement of the recruiting investigator
- Currently participating in any interventional study or clinical trial, or previous participation in an interventional trial, that would render them unsuitable for participation in this trial in the opinion of the recruiting investigator
- Evidence of clinical or electrophysiological neuropathy based on documented medical history and the opinion of the recruiting investigator.

|  | ***R^2^*** |  | ***p value*** |  |
| --- | --- | --- | --- | --- |
| ***Test*** | ***Baseline*** | ***Follow up*** | ***Baseline*** | ***Follow up*** |
| Manual Muscle Testing (Right) (Dorsiflexion) | 0.25 | 0.012 | 0.253 | 0.814 |
| Manual Muscle Testing (Left) (Dorsiflexion) | 0.077 | 0.001 | 0.547 | 0.851 |
| Manual Muscle Testing (Right) (Plantarflexion) | 0.161 | 0.012 | 0.372 | 0.814 |
| Manual Muscle Testing (Left) (Plantarflexion) | 0.03 | 0.001 | 0.706 | 0.851 |
| Grip strength (Right) (kg) | 0.001 | 0.021 | 0.875 | 0.757 |
| Grip strength (Left) (kg) | 0.082 | 0.001 | 0.534 | 0.935 |
| Single Leg Stance (Right) (s) | 0.196 | 0.612 | 0.32 | 0.036 |
| Single Leg Stance (Left) (s) | 0.077 | 0.627 | 0.547 | 0.034 |
| Sit to Stand (s) | 0.002 | 0.015 | 0.933 | 0.792 |

**Supplementary table 1:** Shows R^2^ and p value from simple linear correlation between measured contraction time (ms) and the physical performance test metric. Statistical significance set at (p < 0.0056) following Bonferroni correction.


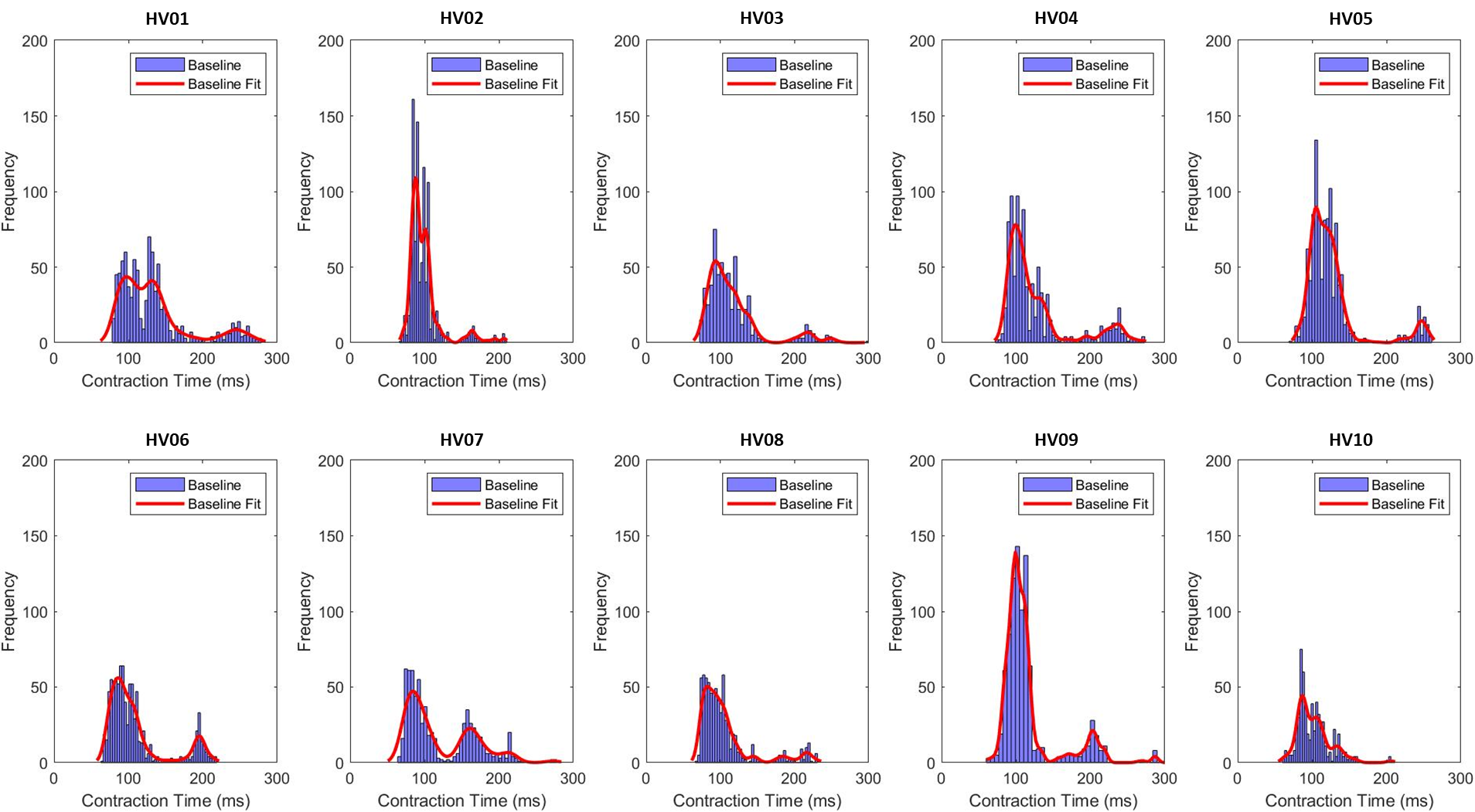


**Supplementary Figure 1:** Histograms of contraction time for each healthy volunteer at baseline on a voxel wise basis. A non-parametric fit is applied to show distribution (red line).
